# Applying the Theory of Constructed Emotion to Police Decision Making

**DOI:** 10.3389/fpsyg.2019.01946

**Published:** 2019-09-11

**Authors:** Joseph Fridman, Lisa Feldman Barrett, Jolie B. Wormwood, Karen S. Quigley

**Affiliations:** ^1^Department of Psychology, Northeastern University, Boston, MA, United States; ^2^Department of Psychiatry, Massachusetts General Hospital, Boston, MA, United States; ^3^Department of Psychology, University of New Hampshire, Durham, NH, United States; ^4^Edith Nourse Rogers Memorial Veterans Hospital, Center for Healthcare Organization and Implementation Research, Bedford, MA, United States

**Keywords:** allostasis, predictive coding, autonomic nervous system, police decision making, theory of constructed emotion, law enforcement

## Abstract

Law enforcement personnel commonly make decisions in stressful circumstances, where the costs associated with errors are high and sometimes fatal. In this paper, we apply a powerful theoretical approach, the theory of constructed emotion (TCE), to understand decision making under evocative circumstances. This theory posits that the primary purpose of a brain is to predictively regulate physiological resources to coordinate the body’s motor activity and learning in the short term, and to meet the body’s needs for growth, survival, and reproduction in the long term. This process of managing the brain and body’s energy needs, called allostasis, is based on the premise that a brain *anticipates* bodily needs and attempts to meet those needs before they arise (e.g., vestibular activity that raises sympathetic nervous system activity *before* standing), because this is more efficient than responding to energetic needs after the fact. In this view, all mental events—cognition, emotion, perception, and action—are shaped by allostasis, and thus all decision making is embodied, predictive, and concerned with balancing energy needs. We also posit a key role for the autonomic nervous system (ANS) in regulating short-term energy expenditures, such that the ANS influences experience and behavior under stressful circumstances, including police decision making. In this paper, we first explain the core features of the TCE, and then offer insights for understanding police decision making in complex, real-world situations. In so doing, we describe how the TCE can be used to guide future studies of realistic decision making in occupations in which people commonly make decisions in evocative situations or under time pressure, such as in law enforcement.

## Introduction

Law enforcement personnel must commonly make high-stakes decisions in stressful circumstances where the costs associated with errors are high and sometimes fatal. The Justice Department found that in 15% of police shootings in Philadelphia over 8 years, the person who was shot was unarmed, but half of these unarmed individuals were perceived to have a weapon (Fachner and Carter, 2015). However, it would be a mistake to assume that decision making errors in stressful contexts are the result of any single feature of a complex situation. Rather, we theorize that decisions made by police officers in highly stressful, time-pressured circumstances are multiply determined by features of the decision making situation, including affectively driven perceptual effects, the current bodily state of the officer, and the officer’s past history or prior experiences in similar contexts.

These high-stakes situations, termed “critical incidents,” often involve witnessing, experiencing, or enacting violent behavior. Officers often describe having distorted perceptions, memory, and thinking both during and after such an incident (Alpert et al., 2012; Novy, 2012). The traumatic distress associated with these critical incidents also can lead to less effective coping and poorer decision making in similar future situations (Cox et al., 2018). Understanding and ultimately improving workplace decision making by personnel who must commonly act under such highly stressful circumstances is important, first and foremost, because their decisions affect the well-being of all those involved. Performance in stressful circumstances can also have important workplace performance and health consequences for law enforcement personnel long after a critical incident (Violanti and Paton, 1999; Franke et al., 2002; Arnetz et al., 2009; Gershon et al., 2009). These same concerns also arise for those in other high-stakes professions, such as military personnel (Johnson et al., 2005; Kavanagh, 2005; Bray et al., 2009; Smith et al., 2011; Rush et al., 2016), or corrections officers (Johnson et al., 2005; Ghaddar et al., 2008; Costello et al., 2015).

To understand and minimize errors in decision making in stressful, complex, real-world contexts, we need innovative experiments and field studies that leverage recent technological advances in ambulatory data collection, including measures of peripheral physiology, cognition, behavioral action, and context (e.g., Andersen and Gustafsberg, 2016; Andersen et al., 2016a,b, 2018). The increasing availability of wearable technologies that can support data collection in more ecologically valid stressful workplace scenarios provides a set of much-needed methodological tools for assessing the physiological changes, real-world behavior, and context of law enforcement officers when they are making mission-critical decisions in the line of duty.

This important translational empirical work must also be guided by current theory. Much of the prior work on decision making in high-stakes workplaces has borrowed from psychological theories derived from experimental evidence gathered in highly controlled, laboratory settings. Such theories often do not translate well to understanding more complex, multifactorial decision making situations, or to a decision-maker who is experiencing strong physiological and subjective arousal in a real-world scenario. In contrast, our recent theory, the theory of constructed emotion (TCE; Barrett, 2017a,b; Hutchinson and Barrett, 2019), can situate the features of multi-factorial, real-world decision making within a single theoretical framework. The TCE integrates evidence from neuroscience, physiology, evolutionary and developmental biology, computational modeling, and engineering to explain how humans construct mental representations (e.g., cognitions, emotions, perceptions, and actions). This framework generates testable hypotheses about how humans will behave and make decisions in real-world scenarios or highly realistic simulations and can guide future work aimed at understanding decision making in stressful occupational settings, like law enforcement. Using this theoretical framework to guide such work has strong potential to result in positive impacts on work performance, health outcomes, and potentially, even to enhance public trust in law enforcement (Jackson, 2015).

Here, we first explain the core features of the TCE, and then illustrate key insights arising from the TCE that are relevant for understanding police decision making in stressful, complex, real-world situations. In so doing, we describe how the TCE can be used to guide future studies of naturalistic decision making in law enforcement or other similar occupations in which people must commonly make decisions under time pressure in stressful or affectively evocative situations.

## The Theory of Constructed Emotion

### Predictive Allostasis: Regulating Energy in the Service of Action and the Role of the Autonomic Nervous System

The theory of constructed emotion (TCE) posits that the primary purpose of an organism’s brain is to coordinate (or regulate) all of the physiological resources required to meet the organism’s imminent needs for action and learning in the short term, and for growth, survival, and reproduction in the long term (Barrett, 2017a,b; Hutchinson and Barrett, 2019). Extensive evidence from the neuroscientific and physiological literatures suggests that energy regulation is best optimized when the brain anticipates bodily needs (Sterling, 2004, 2012); it is more energetically efficient to prepare to meet anticipated needs than to wait and respond to needs after they arise (e.g., if your brain is going to stand you up, the vestibular system increases sympathetic nervous system activity before you stand, lest you faint, which would be energetically costly). This process of predictively managing energy needs is called *allostasis* (Sterling, 2004, 2012; Sterling and Laughlin, 2015). All activities of allostatic regulation—resource acquisition, allocation, and utilization—are posited to operate on a predictive basis to enhance metabolic efficiency. From an organism-level view, we theorize that all mental events—cognition, emotion, perception, and action—are predictive and subject to the constraints of allostasis. As a result, all decision making is embodied, predictive, and fundamentally dependent on how our brains anticipate energy needs.

For the brain to regulate the body, and for the body to maintain support for the brain’s energy needs, there is bi-directional communication between the brain and the systems of the body (i.e., everything outside the brain). The brain sends control messages to organs in the periphery (referred to as efferent signals), and also receives messages from peripheral physiological systems [e.g., afferent signals from organs innervated by or influenced by the autonomic nervous system (ANS), the endocrine system (hormones), and the immune system], which indicate the current state of the body outside the brain (Cacioppo and Berntson, 2011). The ANS, the endocrine system and the immune system all play particularly critical roles in energy regulation because they are the systems that most quickly and directly marshal oxygen, glucose, and other necessary energetic mediators to tissues where they are needed (Bray, 1986). The ANS, which can change organ function on the order of milliseconds to seconds to support imminent action, is especially critical in supporting decision making and related behaviors under stressful circumstances and time pressure. Thus, the ANS is a critical short-term mediator of how the brain achieves allostasis and supports human decision making and action.

### The Brain Creates an Internal Predictive Model to Achieve Allostasis

Recent neuroscientific evidence and computational modeling both converge on the idea that to maintain allostasis, a brain constructs an internal predictive model of the world, and this model includes its own body (Pezzulo et al., 2015; Sterling and Laughlin, 2015; Seth and Friston, 2016; Corcoran and Hohwy, 2017; Barrett, 2017a; Hutchinson and Barrett, 2019). From this perspective, sometimes termed predictive coding (Clark, 2013), Bayesian inference (Deneve, 2008), active inference (Friston, 2010), or predictive processing (Hutchinson and Barrett, 2019), the brain does not simply react to incoming sensory inputs from the world (or from the body); rather it anticipates these inputs by constructing a model of its body in the world. Mental events (i.e., cognitions, emotions, perceptions, and actions) arise from the dynamics of the brain’s “predictions” about the assumed causes of sensory events. These predictions are constantly checked against incoming sensory input (“prediction error”). When prediction error is sufficiently large, the brain updates its internal model, which results in more accurate predictions for similar future situations. The brain aims to construct a model of itself and the body in the world so that fewer unexpected events are encountered in the world, minimizing adverse effects on the organism’s short- and long-term allostasis.

### Affect Is Central to the Brain’s Internal Model

**Figure 1:**
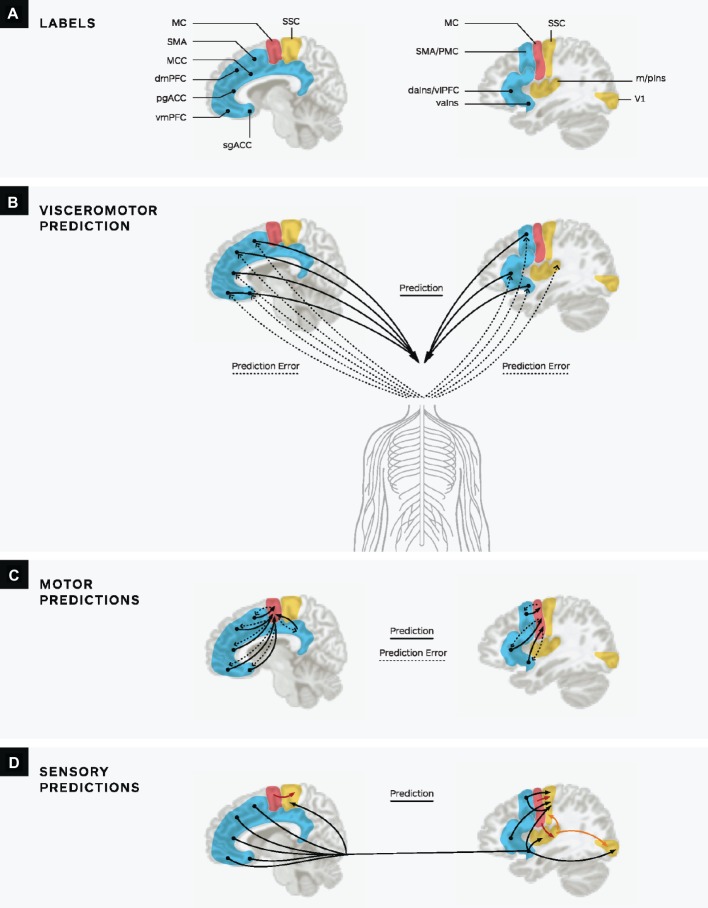
Reproduced with permission of Oxford University Press from Barrett (2017b). A depiction of predictive coding in the human brain. **(A)** Key limbic and paralimbic cortices (in blue) provide cortical control of the body’s internal milieu. Primary MC is depicted in red, and primary sensory regions are in yellow. For simplicity, only primary visual, interoceptive, and somatosensory cortices are shown; subcortical regions are not shown. **(B)** Limbic cortices initiate visceromotor predictions to the hypothalamus and brainstem nuclei (e.g., PAG, PBN, nucleus of the solitary tract) to regulate the autonomic, neuroendocrine, and immune systems (solid lines). The incoming sensory inputs from the internal milieu of the body are carried along the vagus nerve and small diameter C and Ad fibers to limbic regions (dotted lines). Comparisons between prediction signals and ascending sensory input result in prediction error that is available to update the brain’s internal model. In this way, prediction errors are learning signals and therefore adjust subsequent predictions. **(C)** Efferent copies of visceromotor predictions are sent to MC as motor predictions (solid lines) and prediction errors are sent from MC to limbic cortices (dotted lines). **(D)** Sensory cortices receive sensory predictions from several sources. They receive efferent copies of visceromotor predictions (black lines) and efferent copies of motor predictions (red lines). Sensory cortices with less well-developed lamination (e.g., primary interoceptive cortex) also send sensory predictions to cortices with more well-developed granular architecture (e.g., in this figure, somatosensory and primary visual cortices, gold lines). For simplicity’s sake, prediction errors are not depicted in panel D. sgACC, subgenual anterior cingulate cortex; vmPFC, ventromedial prefrontal cortex; pgACC, pregenual anterior cingulate cortex; dmPFC, dorsomedial prefrontal cortex; MCC, midcingulate cortex; vaIns, ventral anterior insula; daIns, dorsal anterior insula and includes ventrolateral prefrontal cortex; SMA, supplementary motor area; PMC, premotor cortex m/pIns, mid/posterior insula (primary interoceptive cortex); SSC, somatosensory cortex; V1, primary visual cortex; and MC, motor cortex (for relevant neuroanatomical references, see Kleckner et al., 2017).

Thirty years of anatomical research (based on tract-tracing studies in primates and other mammals) (Barbas, 2015) indicates that cortical limbic regions are at the top of the brain’s predictive hierarchy (Barrett and Simmons, 2015; Chanes and Barrett, 2016; Barrett, 2017a,b). These regions, which include portions of the cingulate cortex, ventral anterior insula, posterior orbitofrontal cortex, and entorhinal cortex, have the least laminar differentiation, meaning that cytoarchitecturally, they are agranular, with larger layers V and VI, no defined layer IV, and undifferentiated layers II and III (Barbas, 2015; Chanes and Barrett, 2016). These regions are hypothesized to issue, or send, predictions to more differentiated granular cortical areas, including primary motor and sensory cortices (Barbas and García-Cabezas, 2015; Barrett and Simmons, 2015; Finlay and Uchiyama, 2015; Chanes and Barrett, 2016), as seen in Figure 1. These same cortical limbic regions both support allostasis (Kleckner et al., 2017) and receive relayed sensory inputs from organs, joints, and skin in the periphery, which is called interoception (Vaitl, 1996; Cameron, 2001; Craig, 2002, 2003a,b, 2009, 2014; Berntson et al., 2018). Interoceptive visceral afferents from organs innervated by or influenced by the autonomic nervous system (ANS), the endocrine system (hormones), and the immune system carry relatively low-resolution information, and we hypothesize that these inputs are experienced as low-dimensional affective feelings (i.e., general feelings that vary in pleasantness/unpleasantness and activation/deactivation; Barrett and Bliss-Moreau, 2009; Barrett, 2017a; Hutchinson and Barrett, 2019). As such, interoceptive sensations from the periphery and their representation as affective feelings are at the core of the neural architecture which issues the brain’s internal model (Craig, 2014; Chanes and Barrett, 2016; Barrett, 2017a,b; Hutchinson and Barrett, 2019). Your brain is always trying to maintain allostasis, and so it is always modeling the interoceptive state of the body. Therefore, affective feelings are a property of consciousness, and affect is at the core of all mental events that your brain constructs.

### Accurate Predictions Require Variability Across Mental Instances

A critical feature enabling a brain to operate predictively is its ability to generalize or create higher level summaries from particulars. That means that the organism must constantly query its existing model about the current sensory array and what it is *most like* from its prior experience. Generalizations, also called abstractions, are constructed as an organism updates its internal model based on a wide range of highly variable instances over time. Predictions issued from limbic cortices to sensory and motor cortices are constructed from the organism’s past experience with similar internal and external contexts. However, these contexts are never exactly the same twice. This means that the brain must quickly generalize from one situation to another even when they are somewhat different, creating *ad hoc* generalizations in the moment to categorize a given instance as more similar to one prior experience than to another. Predictions underlying particular instances of cognitions, emotions, perceptions, and actions will, therefore, be highly context-bound. Because predictions are context-bound, and the contexts in which humans find themselves can vary widely, we expect there to be substantial variability across *instances* of experience within a person, even when the instances are categorized and labeled as belonging to the same *category* of experience (e.g., anger). Instances labeled with the same category will have variable features—including affective features, physiological features, action or movement-related features, and brain features. For example, fear in the context of taking a rollercoaster ride has different features than fear in the context of hearing footsteps following you while walking in a dark alley. Both the common and the divergent features across each of the variable instances of a category are hypothesized to become part of the generalization (or abstraction) for that category as constructed by the individual as part of their internal model. The internal model will reflect that some subset of the features of one instance of the category will be similar across instances, but that the exact subset of features, including contextual features, will vary across different instances labeled as belonging to the same category.

The nature and extent of this immense variability has been a significant point of contention, for example, among theorists who disagree about whether the variability in features that is observed among instances labeled as belonging to the same emotion category is meaningful (as the TCE suggests) or simply random error (as other theories posit). Emotions traditionally were, and in some quarters still are, commonly assumed to have an essence, meaning that all instances of a given emotion are presumed to have a core similarity either at a neural or physiological level (Barrett, 2006, 2017a,b). However, empirical evidence reveals a striking lack of consistency in emotional experience and expression, such that specific emotional instances (e.g., an experience categorized as anger or fear in a specific time and place) are highly variable across contexts, even within a person. Empirically, there are no biological “fingerprints” for specific emotion categories in the brain (Lindquist et al., 2012; Clark-Polner et al., 2017), in the face (Barrett et al., 2019), in the body (Siegel et al., 2018a), or in experience (Lindquist et al., 2013). That is, an emotion does not have unique and consistent physical features across individuals, or even within the same individual across instances. Thus, quite distinct experiences can be categorized (or labeled) as belonging to the same emotion category but still vary considerably in their features (e.g., whether anger is associated with a heart rate increase or decrease, or whether it is associated with a scowl on the face or not). Likewise, very similar affective experiences can be categorized and labeled as belonging to different emotion categories across instances (Lindquist et al., 2013). For example, it can be difficult to tell if you are feeling fear or excitement in advance of an important event (e.g., a marriage, the birth of a child, a major sporting event of your favorite team). According to the TCE, and consistent with decades of empirical evidence, variability within and across instances of different emotion categories (including distress) is the norm, and this variability is functionally important and relevant for allostasis, because allostatic needs will vary across situations that have different energetic consequences even for instances labeled as the same emotional or affective experience.

## Novel Implications From the Theory of Constructed Emotion for Decision Making in Law Enforcement

In this section, we use law enforcement examples to demonstrate how the TCE offers a unique perspective from which to examine decision making under stress among law enforcement personnel. The TCE offers several key insights that may shed new light on energetic, brain, and/or bodily processes that underlie decision making under stressful circumstances or that could be leveraged to design interventions to improve decision making in such contexts. We provide examples of (1) how allostasis and affective feelings predictively shape perception and action; (2) the central role of interoception and related peripheral physiological arousal in cognition, emotion, perception, and action; and (3) the role of individual differences and variability in cognition, emotion, perception, action, and thereby, decision making in law enforcement settings.

### The Role of Affective Feelings in Predictively Shaping Perception and Action

Between 2007 and 2014, 49% of “officer-involved shootings” in Philadelphia in which the victim was unarmed were attributed to “threat perception failures,” meaning a non-threatening object or movement led the officer to perceive the subject as being armed (Fachner and Carter, 2015). Despite having roughly the same population of White and Black citizens, 80% of the suspects in officer-involved shootings in Philadelphia during this time period were Black, with the US Justice Department reporting that Black suspects were most likely to be the subject of a threat perception failure (Fachner and Carter, 2015). This is consistent with other findings showing that individuals from racial minority groups experience more force in encounters with police (Fryer, 2016), are treated less respectfully during traffic stops (Voigt et al., 2017), are more likely to be shot at, even when unarmed (Brown and Langan, 2001; Ross, 2015), and that further, these effects remain even when suspect behavior is controlled for (Scott et al., 2017). This is also consistent with other empirical work demonstrating that social categories like race have a pronounced impact on threat perception (Payne, 2001; Correll et al., 2002, 2007; Eberhardt et al., 2004; Payne et al., 2005), and that affective feelings are integral to how we perceive and respond to others across a range of social categories (Cuddy and Fiske, 2002; DeSteno et al., 2004; Russell and Fiske, 2008). The racial and cultural stereotypes thought to underlie these biases in threat perception are imbued with affective features, and some data suggest that more general beliefs concerning the likelihood of encountering interpersonal threats may partially explain race-based shooter biases, even in the absence of racial or cultural stereotypes (Miller et al., 2012).

The TCE, at its heart, posits that brains use predictive processing in the service of optimizing energy regulation. The agranular limbic brain areas involved in implementing allostasis and the experience of affect are both central to energy regulation, and anatomically speaking, reside at the top of the brain’s predictive hierarchy. As a result, affect and affective predictions critically shape perception and behavior (see, e.g., Chanes et al., 2018). In previous research, we have proposed that you “perceive what you feel,” a phenomenon we have termed “affective realism” (Anderson et al., 2011, 2012; Barrett and Wormwood, 2015; Wormwood et al., 2018; Siegel et al., 2018b). This means that affective experience critically shapes what we *expect to* and *actually do* see, hear, and smell. In this way, affect infuses all perception and action, including decision making in the time-pressured, stressful situations encountered by police officers.

We have demonstrated that both experimental affective inductions and naturally occurring evocative situations evoking intense affect can significantly shape perceptions of social and threat-related information (Baumann and DeSteno, 2010; Wormwood et al., 2016, 2017, 2019; Siegel et al., 2018b). For example, individuals induced to experience an instance of anger were more likely to exhibit biased perceptual decision making in a gun detection task, such that they were more likely to make misidentification errors “seeing” unarmed individuals as armed than vice versa (Baumann and DeSteno, 2010). Critically, this biased perception was causally explained by anger’s influence on predictions: angry participants expected to encounter more armed suspects, and controlling for these expectancies mitigated the impact of anger on threat perception. In another experiment, participants in Boston tested about 1 month after the Boston Marathon bombings completed a threat perception task where they attempted to shoot armed targets and avoid shooting unarmed targets (Wormwood et al., 2016). Prior to the threat perception task, participants viewed images taken from news coverage of the bombings that were either accompanied by threat-related headlines (e.g., “Not Since 9/11”) or more affectively positive headlines focused on the community’s resilience following the attack (e.g., “Boston Strong”). Participants made more errors shooting unarmed targets if they had viewed the negatively framed terror attack images than if they viewed the positively framed images. Critically, we showed that the observed increase in shooting of unarmed targets was caused by decreased perceptual sensitivity (i.e., a reduced ability to distinguish targets holding a gun from those who were not), and perceptual sensitivity was also impacted by how strongly the participant reported having been affected by the bombings when they occurred.

Taken together, theoretical and empirical work suggest that feeling significantly distressed or threatened can predictively contribute to perceiving the world as more stressful or threatening in a very literal sense. This work also suggests that an officer’s experience of danger or threat can be “affectively real” even when the situation is non-threatening in other ways (i.e., there is no weapon). Affective realism also has implications for how bystanders think about misperceptions in police decision making: when feeling threatened or affectively aroused, officers could “see” a gun in the hand of a suspect or perceive a suspect as behaving aggressively whereas someone who is not feeling threatened or not affectively aroused would not. This is not a *post hoc* justification for errant behavior, but the perception of danger can be objectively incorrect even while being “affectively real.” This perspective strongly implies that police officer trainings should (1) help police officers learn to be more attentive to their bodily feelings, (2) help officers recognize when their perceptions could be shaped by their predictions, and (3) help them learn to consider alternative interpretations before a situation becomes a critical incident or a critical incident becomes even more dire.

### Interoception Predictively Shapes Cognition, Emotion, Perception, and Action

Consider a police officer nearing the end of a busy, stressful 12-h patrol shift. The officer steps back into their cruiser after drinking two cups of coffee when a dispatch call comes in about a nearby prowler. Driving over to investigate, the officer’s heart is racing, palms sweating, stomach clenching, and face flushing. Minutes later, in the same neighborhood, the officer encounters a teenage loiterer on the phone, who, when seeing the officer, scowls and turns away. The officer loudly instructs the teenager to end the call, turn around, and answer some questions. From the perspective of the TCE, the officer’s internal sensations of greater peripheral physiological arousal predictively shape their experience and actions in this situation, and do so regardless of whether the officer is consciously aware of these sensations. If the officer is not consciously aware of these changes—in this case a quickly beating heart, sweating, clenched stomach, and facial flush—this physiological arousal may instead be incorporated into the officer’s internal model of the current situation as threatening (i.e., the heightened bodily arousal shapes affective predictions concerning the presence or likelihood of threats that would necessitate the current bodily arousal, directing perception and action accordingly). In fact, those sensations are internal to the officer and very likely created by other features of the context: the two cups of coffee just consumed, the recently received call to be on the lookout for a prowler, and the rest of the officer’s day having been busy and stressful. The officer who is unaware of the possible range of sources for these feelings is very likely to construe the situation with the loitering teenager as tense, the teenager as surly, and as a result, the officer might act harshly. The TCE highlights the importance of making police officers aware of the ways in which internal sensations, which can arise from many different sources, can color our experience and our actions. Indeed, several of the newest officer training protocols specifically make officers more aware of their physiological arousal by enabling them to measure and track it over time using wearable devices, and then training officers to use strategies to reduce this arousal (Arnetz et al., 2009, 2013; Andersen and Gustafsberg, 2016; Andersen et al., 2018).

The peripheral physiological systems that are responsible for feeling aroused or activated, most notably the cardiovascular, respiratory, and gastrointestinal systems, are the very same systems that support attainment and movement of energetic resources (such as oxygen and glucose) around the body to where they are needed to enact allostasis. Indeed, we meet most of our most basic short- and long-term allostatic needs by moving the body—this includes respiration, consumption (of water and nutrients), communication, copulation, and ambulation and associated changes in posture. Thus, allostasis requires action planning, which itself requires very context-specific assessments of predicted metabolic needs and environmental affordances that must be tightly coupled with changes in peripheral physiological activity which prepare for and enable these upcoming actions. The idea that the cardiovascular system operated predominantly in the service of action was earlier posited by Paul Obrist and colleagues, as the concept of “cardio-somatic coupling” such that cardiac activity changed in advance of expected action (Obrist et al., 1969, 1970). From this perspective, we propose that future research aimed at improving police decision making under stressful situations would benefit from robust measurement of physiological changes during both training scenarios as well as real-world law enforcement situations. Tracking these measures may help officers learn to attend to the myriad possible sources of physiological activation and feelings of arousal so that they can consider alternative explanations for the felt arousal. Police training that incorporates peripheral physiological measures and can demonstrate these effects for officers may help make very real the importance of such bodily changes in a law enforcement context.

Future research is needed to examine how allostasis and interoception together create mental experiences, especially in dynamically changing real-world contexts. Although technologies enabling the collection of affective, physiological, or contextual information could be useful as training feedback, there are obstacles to deploying self-monitoring technologies in the field. For one, despite the increasing prominence of body camera surveillance and calls within the profession to implement biometric monitoring of police officers (Bedford, 2019), there is still reticence among first-responders to continuous monitoring of their physiology, perhaps because such initiatives are still nascent and not in common use. Others are unconvinced that the benefits of such sociotechnical tools outweigh potential accompanying downsides which could negatively impact decision making and performance—for example, inadvertently inducing anxiety or panic in officers or distracting from the processing or awareness of critical exteroceptive sensory information. Still, some researchers have been able to build collaborative partnerships with police officers to test the use of techniques like portable heart rate variability biofeedback to reduce lethal force errors (see, e.g., Andersen et al., 2018).

Another key feature of these bodily sensations that is highlighted by the TCE framework (and noted above) is that the sensory signals from the organs, joints, and skin of the body (interoceptive signals; Craig, 2002, 2003a,b, 2009) have lower fidelity (low signal-to-noise ratio) than those coming from the exteroceptive senses (Barrett and Simmons, 2015; Chanes and Barrett, 2016; Khalsa et al., 2018; Hutchinson and Barrett, 2019). We propose that the low fidelity of interoceptive signals is a key reason why it can be difficult to pinpoint the source or meaning of low-dimensional affective feelings, which are proposed to be the experiential sequelae of interoceptive signals. These diffuse interoceptive signals provide a source of prediction error to the brain, which can alter the brain’s internal predictive model, and in turn generate new predictions.

Interoceptive signaling from the heart *via* the baroreceptors has been shown to be one interoceptive signal influencing behavior and action. Interoceptive information from the heart arrives in phases because the pressure-sensitive baroreceptors fire at systole, when the heart is ejecting blood into the aorta, and are less active during diastole. This phenomenon provides a unique opportunity for exploring state-related changes in affectively mediated prediction because researchers can synchronize the presentation of exteroceptive stimuli with systole or diastole to vary the interoceptive context concurrent with a constant exteroceptive stimulus. Time-locked presentation of stimuli with cardiac interoceptive information can enhance or inhibit aspects of perception, memory, and action. For example, baroreceptor stimulation reliably decreases pain ratings (Dworkin et al., 1994). Likewise, Garfinkel et al. (2013) have shown that memory for target words presented at high speeds suffered from interference when presented during systole compared to those presented during diastole, whereas detection, learning, and even exposure therapy for fearful stimuli are enhanced by presentation of stimuli at systole (Garfinkel et al., 2014; Pfeifer et al., 2017; Watson et al., 2019). This paradigm has been adapted to show that race-driven misidentifications of threat during a weapons identification task and a first-person shooter task were significantly increased by presentation during systole (Azevedo et al., 2017). These findings demonstrate the importance of developing methodologies to investigate the influence of particular interoceptive contexts on perception and action.

Critically, there are striking individual differences in sensitivity for detecting internal sensations from the body (i.e., Jones, 1994; Knapp et al., 1997; Barrett et al., 2004), or interoceptive sensitivity. Further, interoceptive sensitivity can be altered using either pharmacological methods (Khalsa et al., 2009; Hassanpour et al., 2018) or environmental stimulation (Feinstein et al., 2018). Thus, individual differences in interoceptive sensitivity could critically underlie differences across people in affective experience and behavior under stressful circumstances. Previous work also shows that interoceptive sensitivity moderates the relationship between peripheral physiological activity and self-reported experience (Barrett et al., 2004; Pollatos et al., 2007; Garfinkel et al., 2014; Pfeifer et al., 2017), such that physiology and experience or behavior are more tightly coupled in those with higher interoceptive sensitivity. In a related study, Dunn and colleagues (Dunn et al., 2010) found that participants’ ability to track their heartbeats was positively correlated with the strength of the association between anticipatory bodily signals (electrodermal activity and heart rate) and decision making in an intuitive reasoning task. (However, the tracking task used in the Dunn et al. study does not permit a strictly interoceptive interpretation of the findings; for a discussion of the limitations of the heartbeat tracking task, see Ring et al., 2015; Desmedt et al., 2018). Further, higher interoceptive sensitivity does not necessarily mean that one is more interoceptively aware of or more likely to notice one’s bodily states (Chentsova-Dutton and Dzokoto, 2014; Garfinkel et al., 2015, 2016; Critchley and Garfinkel, 2017). Thus, individual differences in this psychological facet of interoception may not reflect individual differences in the strength or patterning of afferent neural signaling coming from the periphery (Critchley and Garfinkel, 2017).

One implication of these findings is that police officers may also benefit from trainings that promote greater awareness of their interoceptive signals. For example, prior empirical work showed that mindfulness interventions may foster long-term increases in an individual’s ability to attend to interoceptive sensations (Farb et al., 2013), and similar interventions have been shown to improve cognitive performance in a sustained attention Go/No Go task in a high-stress military cohort (Jha et al., 2017). Recent exploratory work with law enforcement personnel suggests that officers would be willing to undertake similar evidence-based training that focused on the effects of distress and trauma (Andersen et al., 2015). These findings suggest that new interventions could target interoceptive sensitivity training for police officers. Finally, we note that augmenting interoceptive awareness may not always be useful, and in some moments or contexts, higher interoceptive awareness could distract from other more pressing incoming sensory information. Thus, one needs to take this issue into account when designing an interoceptive intervention.

### The Role of Individual Differences and Variability in Cognition, Emotion, Perception, and Action

To execute physical feats in stressful situations, professionals in fields such as car racing, civil aviation, medicine, and law enforcement now commonly train using realistic, and sometimes stressful, situations. Although classroom preparation can help professionals to understand work goals, interpret training feedback, and learn some tasks, no one can learn to drive a race car, do surgery, land a plane, or become a police officer just by reading or talking about it. It takes experience to detect and assess goal-relevant signals in perceptually complex environments, to know which strategy to deploy to mitigate the chances of catastrophic failure, and to successfully complete physically demanding tasks under highly time-pressured or stressful circumstances.

The TCE specifically addresses how context-specific work-oriented predictions can drive experience and behavior. In the case of police officer training, the TCE emphasizes the importance of recognizing that each instance or experience of “distress” can be quite different from every other one, and that sensations from the body also can vary considerably depending on the affective or performative (action-oriented) context of the situation. Because officers will experience a wide range of stressful situations in the field, including critical incidents, the TCE suggests that trainings must also occur across a range of diverse and highly realistic scenarios, which we propose would hasten the process of gaining needed experience and reduce the chances of real-world errors. Consistent with this perspective, a study by Arnetz et al. (2009) using realistic training scenarios provided evidence of better performance, less negative mood, less cardiovascular reactivity, and less distress in those who received real-world, in-the-moment training (termed resilience training by these authors), than in those who did not take this training (Arnetz et al., 2009). Such training also can enable an officer to focus attention on important features of the external environment and, at the same time, reduce physiological arousal that could lead to poor decisions.

Engaging in more realistic and variable training scenarios also should better prepare police officers to recognize, and potentially overcome, affective realism effects in the case of biased perceptions of threat under stressful conditions in the field. To be maximally effective, these trainings must reliably induce affective experiences with similar potency to those they may experience on the job, which can be assessed using ambulatory technology. Because prior research has demonstrated that affective feelings can shape not only actions in a threat perception task (Wormwood et al., 2016), but perceptions of social others as well (Cuddy and Fiske, 2002; DeSteno et al., 2004; Russell and Fiske, 2008), we recommend that these training situations also involve learning to consider potential alternative interpretations of others’ affective states and intended actions, especially in highly evocative contexts. Officers can also be trained to consider alternative interpretations of their own affective states, which in some theoretical perspectives would be called emotion regulation, but in the TCE is simply part of the process of creating another emotional experience (see Gross and Feldman Barrett, 2011 for further discussion).

These training scenarios should also utilize other people, including other officers, to increase the realism, unpredictability, and variability in the behaviors observed in the scenarios. Inclusion of team members within the same training scenarios also can enhance cohesion especially among team members who have little prior experience with one another. Organizational science has consistently documented that building social cohesion is critical to effective teamwork (e.g., NTSB, 1994), particularly at a new worksite, or when the composition of the team is changed (Huckman and Pisano, 2006; Groysberg et al., 2008). These findings are also consistent with proposals of the TCE that unfamiliarity with or lack of predictability in the behavior of social others may lead to errors in prediction (Theriault et al., 2019), which we suggest could be corrected through ecologically valid, team-based training.

## Conclusion

According to Barrett (2017a,b), a person’s *affective niche* comprises whatever “objects and events will impact [their] body budget, changing [their] affect” (Barrett, 2017a, p. 73). Our affective niche is shaped by the state of our bodies and our expectations about our bodies. These expectations are informed by our past experiences and our culture, which includes our prior work experiences. The affective niche of police officers and other first responders are rich and complex, involving civilians and suspects, superiors and colleagues, and a wide variety of stressful and potentially traumatic situations or events. Police officers also have to consider how their performance will be evaluated by the public, governmental groups that set policy, oversight bodies, and the media. Despite the relatively large and rich affective niche of police officers, there are strong professional pressures to avoid attending to or conceptualizing sensations as feelings or emotions, or what one survival guide for officers called the “biological rollercoaster” of policing (Gilmartin, 2002). Drodge and Murphy (2002) noted: “[i]t is ironic that police personnel are socialized to curb their own emotions, and there are strong cultural norms aimed at controlling this, whereas on the other hand, they are trained to be vigilant about other people’s emotional displays, particularly criminal suspects” (Drodge and Murphy, 2002, p. 426). This report and others also describe how these norms can have long-term negative mental and physical effects on police officers and their families (Pogrebin and Poole, 1991; Galatzer-Levy et al., 2013). Some of the physical effects could arise as sequelae of the repeated autonomic nervous system and endocrine system activation that occurs over a work career characterized by frequent, stressful situations (Planche et al., 2019).

Police officers also are at increased risk for more distal possible health consequences (e.g., cardiovascular disease, links between PTSD & cardiovascular health risk, metabolic syndrome, obesity) than those in less stressful occupations (Violanti et al., 2006a,b). Many of these chronic health conditions are characterized by allostatic dysregulation and are exacerbated by police work schedules that disrupt circadian rhythms (Vila et al., 2000; Vila, 2006; Violanti et al., 2009). Critical incident exposure specifically has been found to be associated with increased occurrence of nightmares and poor global sleep quality (Neylan et al., 2002). Additionally, poor sleep in police officers mediated the relationship between traumatic stress symptoms and health functioning (Mohr et al., 2003). Finally, even though police suicides tend to be under-reported (Violanti et al., 1996), police officers are significantly more likely than the general public to die of suicide (Violanti et al., 1998), with more police officers dying of suicide in the past 3 years than in the line of duty (Lohr, 2019). In short, police officers bear major chronic health burdens that we propose are due in part to allostatic dysregulation that could be mitigated by training that focuses more on attending to, and learning to reduce, the physiological arousal that commonly occurs in the law enforcement workplace.

The TCE offers a unique perspective from which to examine the affective niche of police officers, along with other first responders, military personnel, and corrections officers, all of whom have similarly stressful occupations. Importantly, our framework suggests there are important neurobiological and energetic mechanisms that support decision making among personnel who must make high-stakes decisions under extremely stressful conditions on a regular basis. The TCE also points to novel future interventions aimed at improving decision making and cognitive performance in stressful situations. Understanding that human brains use predictive processing to enact allostasis and create cognition, emotion (including affective feelings), perception, and action will not only lead to better models of individual behavior across contexts, but to better training regimens and other occupational interventions to reduce negative health outcomes for police officers. Applying findings from contemporary affective science to policing could improve the accuracy of police decision making in stressful contexts and increase the mental and physical resilience of law enforcement officers.

## Author Contributions

All authors contributed to manuscript drafting and revision, and read and approved the submitted version.

### Conflict of Interest Statement

The authors declare that the research was conducted in the absence of any commercial or financial relationships that could be construed as a potential conflict of interest.
